# Glasgow prognostic score is a better predictor of the long-term survival in patients with gastric cancer, compared to the modified Glasgow prognostic score or high-sensitivity modified Glasgow prognostic score

**DOI:** 10.18632/oncotarget.27796

**Published:** 2020-11-10

**Authors:** Noriyuki Hirahara, Takeshi Matsubara, Shunsuke Kaji, Yasunari Kawabata, Ryoji Hyakudomi, Tetsu Yamamoto, Yuki Uchida, Kazunari Ishitobi, Kiyoe Takai, Yoshitsugu Tajima

**Affiliations:** ^1^Department of Digestive and General Surgery, Shimane University Faculty of Medicine, Shimane, Japan

**Keywords:** glasgow prognostic score (GPS), modified GPS (mGPS), high-sensitivity mGPS (HS-mGPS), C-reactive protein, gastric cancer

## Abstract

Background: Inflammation influences cancer progression by increasing catabolism and impairing nutrient absorption. We compared the prognostic ability of three inflammation-based prognostic scoring systems—the Glasgow prognostic score (GPS), modified GPS (mGPS), and high-sensitivity mGPS (HS-mGPS)—in gastric cancer patients.

Materials and Methods: We retrospectively examined 434 curatively resected gastric cancer patients to evaluate the prognostic ability of scoring systems for overall survival (OS) and cancer-specific survival (CSS).

Results: OS analysis identified the following independent prognostic factors: GPS model: pathological stage (pStage, *p* < 0.001), carcinoembryonic antigen (CEA, *p* = 0.004), and GPS 1 (hazard ratio [HR], 1.929; 95% confidence interval [CI], 1.152-3.228; *p* = 0.013); mGPS model: body mass index (BMI, *p* = 0.027), pStage (*p* < 0.001), and CEA (*p* < 0.001); HS-mGPS model: BMI (*p* = 0.029), pStage (*p* < 0.001), and CEA (*p* = 0.003). mGPS and HS-mGPS were not independent prognostic factors for OS. CSS analysis of the GPS model identified pStage (*p* < 0.001), CEA (*p* = 0.015), and GPS 1 (HR; 2.095, 95% CI; 1.025–4.283; *p* = 0.043) and 2 (HR, 2.812; 95% CI, 1.111–7.116; *p* = 0.029) as independent prognostic factors; however, mGPS and HS-mGPS were not independent prognostic factors for CSS. Log-rank tests demonstrated significant differences in OS among patients with GPS 0 vs. 1 (*p* < 0.001) and 0 vs. 2 (*p* < 0.001) and in CSS among the three GPS (0 vs. 1; *p* = 0.005, 0 vs. 2; *p* < 0.001, 1 vs. 2; *p* = 0.009).

Conclusions: GPS most reliably predicts long-term survival of gastric cancer patients.

## INTRODUCTION

Nutrition and inflammation are closely related; inflammation induces malnutrition by increasing catabolism and impairing nutrient absorption, and conversely, malnutrition promotes the severity of inflammation [1, 2].

C-reactive protein (CRP) is a sensitive marker of an infectious or inflammatory disease state and is also an important hallmark of carcinogenesis and cancer progression [3, 4]. The Glasgow prognostic score (GPS) is evaluated using serum CRP and albumin levels. It was first proposed as a prognostic indicator in patients with unresectable lung cancer [5], and its prognostic significance has been validated in different types of cancers [6–10]. The modified GPS (mGPS) highlights the importance of CRP; if CRP is elevated, even patients with normal albumin levels are assigned a score of 1 [11]. Presently, the mGPS is more widely used than the GPS to assess the prognoses of cancer patients [12–14]. Notably, the high-sensitivity mGPS (HS-mGPS) places a higher weightage on the CR*P* value by setting the cut-off CR*P* value to 0.3 mg/dl, to further enhance the prognostic value of the mGPS [15].

The utility of preoperative evaluation by using GPS and mGPS for estimating the prognoses in patients with various cancers has been demonstrated in several studies [5–14]. However, the impact of HS-mGPS on the long-term outcomes of patients with gastric cancer has not been fully elucidated. Here, we evaluated the prognostic impact of HS-mGPS, and compared the prognostic abilities of the GPS, mGPS, and HS-mGPS in patients with gastric cancer who underwent gastrectomy.

## RESULTS

### Receiver operating characteristic (ROC) curves of inflammation-based prognostic scores

**Figure 1:**
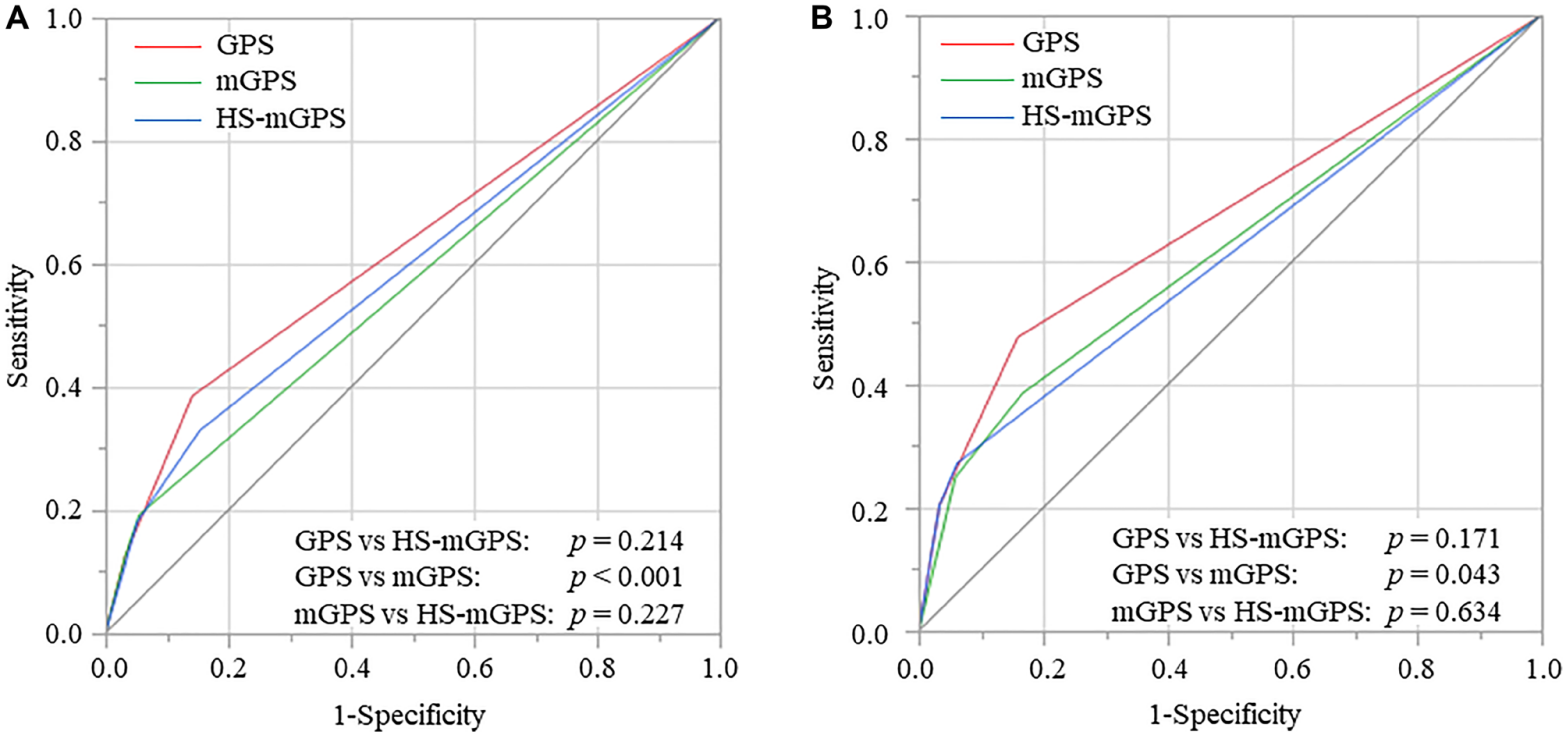
Receiver operating characteristic curves of GPS, mGPS, and HS-mGPS for OS (**A**) and CSS (**B**), GPS, Glasgow prognostic score; mGPS, modified GPS; HS-mGPS, high-sensitivity mGPS; OS, overall survival; CSS, cancer-specific survival.

The area under the curve (AUC) values of the GPS, mGPS, and HS-mGPS for predicting overall survival (OS) were 0.625, 0.570, and 0.593, respectively. The GPS had the greatest AUC value among the three inflammation-based prognostic scoring systems, and this value was significantly higher than the corresponding value for the mGPS (*p* < 0.001) (Figure 1A).

The AUC values of the GPS, mGPS, and HS-mGPS for predicting cancer-specific survival (CSS) were 0.667, 0.607, and 0.619, respectively. The AUC value of the GPS was significantly higher than the corresponding value for the mGPS (*p* = 0.043) (Figure 1B).

### Clinicopathological characteristics and inflammation-based prognostic scores

The baseline characteristics of the 434 patients with gastric cancer and their associations with the GPS, mGPS, and HS-mGPS are shown in Supplementary Table 1. Based on the GPS, 351 (80.9%), 61 (14.1%), and 22 (5.1%) patients were assigned scores of 0, 1, and 2, respectively. The GPS was significantly associated with age (*p* = 0.005), body mass index (BMI, *p* = 0.010), albumin (*p* < 0.001), CRP (*p* < 0.001), operative procedure (*p* = 0.013), tumor size (*p* < 0.001), tumor depth (*p* < 0.001), lymph node metastasis (*p* = 0.023), pathological Tumor-Node-Metastasis or pTNM stage (*p* < 0.001), and postoperative complications (*p* < 0.001).

The mGPS was allocated as follows: scores 0, 1, and 2 in 398 (91.7%), 14 (3.2%), and 22 (5.1%) patients, respectively. The mGPS was significantly associated with age (*p* = 0.021), albumin (*p* < 0.001), CRP (*p* < 0.001), operative procedure (*p* = 0.021), tumor size (*p* < 0.001), tumor depth (*p* < 0.001), lymph node metastasis (*p* = 0.015), pTNM stage (*p* < 0.001), carcinoembryonic antigen (CEA) (*p* = 0.048), and postoperative complications (*p* < 0.001).

The HS-mGPS of 0, 1, and 2 was assigned to 352 (81.1%), 48 (11.1%), and 34 (7.8%) patients, respectively. The HS-mGPS was significantly associated with clinicopathological factors, such as age (*p* = 0.022), albumin (*p* < 0.001), CRP (*p* < 0.001), operative procedure (*p* = 0.007), tumor size (*p* < 0.001), tumor depth (*p* < 0.001), lymph node metastasis (*p* = 0.017), pTNM stage (*p* < 0.001), and postoperative complications (*p* < 0.001).

### Validation of inflammation-based prognostic scores for OS

Variables with *p*-values of < 0.05 in univariate analyses were subjected to multivariate analysis. In the multivariate analysis of the GPS model, pathological stage or pStage (hazard ratio [HR], 2.817; 95% confidence interval [CI], 1.638–4.844; *p* < 0.001), CEA (HR, 1.942; 95% CI, 1.239–3.044; *p* = 0.004), and GPS 1 (HR, 1.929; 95% CI, 1.152–3.228; *p* = 0.013) were identified as independent prognostic factors for OS. In the mGPS model, BMI (HR, 1.942; 95% CI, 1.077–3.504; *p* = 0.027), pStage (HR, 2.747; 95% CI, 1.583–4.766; *p* < 0.001), and CEA (HR, 1.958; 95% CI, 1.248–3.070; *p* < 0.001) were identified as independent prognostic factors for OS. However, an mGPS of 1 and 2 identified as significant indicators of unfavorable OS (*p* = 0.021 and *p* < 0.001, respectively) in univariate analyses were not confirmed to be independent prognostic factors (*p* = 0.062 and *p* = 0.228, respectively) in multivariate analysis. In the HS-mGPS model, BMI (HR, 1.923; 95% CI, 1.070–3.459; *p* = 0.029), pStage (HR, 2.890; 95% CI, 1.680–4.971; *p* < 0.001), and CEA (HR, 1.963; 95% CI, 1.253–3.076; *p* = 0.003) were identified as independent prognostic factors for OS. An HS-mGPS of 2 identified as a significant indicator of unfavorable OS (*p* < 0.001) in univariate analyses was not confirmed to be an independent prognostic factor (*p* = 0.155) in multivariate analysis (Table 1).

**Table 1:** Univariate and multivariate analyses of clinicopathological factors affecting OS after laparoscopic gastrectomy for gastric cancer

	Variables	Univariate analysis			Multivariate analysis with GPS			Multivariate analysis with mGPS			Multivariate analysis with HS-mGPS		
		HR	95% CI	*p* value	HR	95% CI	*p* value	HR	95% CI	*p* value	HR	95% CI	*p* value
Age	< 70	1.000											
	≥ 70	1.617	1.044–2.505	0.032	1.175	0.730–1.892	0.506	1.202	0.747–1.934	0.448	1.236	0.771–1.982	0.378
Gender	female	1.000											
	Male	1.604	0.974–2.642	0.063									
BMI	≥ 18.5	1.000											
	< 18.5	1.905	1.075–3.375	0.027	1.723	0.950–3.126	0.073	1.942	1.077–3.504	0.027	1.923	1.070–3.459	0.029
Tumor size	< 5	1.000											
	≥ 5	2.334	1.525–3.573	< 0.001	1.108	0.636–1.930	0.716	1.238	0.709–2.162	0.453	1.135	0.645–1.996	0.661
Diff.	well & mod	1.000											
	Poor	1.656	1.087–2.522	0.019	1.459	0.922–2.308	0.107	1.427	0.901–2.261	0.130	1.426	0.904–2.251	0.127
pSstage	1 & 2	1.000											
	3	4.000	2.612–6.125	< 0.001	2.817	1.638–4.844	<0.001	2.747	1.583–4.766	< 0.001	2.890	1.680–4.971	< 0.001
CEA	< 5.0	1.000											
	≥ 5.0	2.350	1.528–3.612	< 0.001	1.942	1.239–3.044	0.004	1.958	1.248–3.070	< 0.001	1.963	1.253–3.076	0.003
GPS	0	1.000											
	1	2.602	1.594–4.245	< 0.001	1.929	1.152–3.228	0.013						
	2	5.240	2.722–10.089	< 0.001	1.887	0.837–4.254	0.126						
mGPS	0	1.000						1.000					
	1	2.681	1.163–6.183	0.021				2.299	0.960–5.505	0.062			
	2	4.041	2.133–7.657	< 0.001				1.631	0.736–3.613	0.228			
HS-mGPS	0	1.000									1.000		
	1	1.568	0.860–2.861	0.142									
	2	3.660	2.103–6.370	< 0.001							1.610	0.835–3.104	0.155
Complications	Absent	1.000											
	Present	1.876	1.220–2.884	0.004	1.445	0.890–2.346	0.136	1.428	0.874–2.331	0.155	1.473	0.912–2.380	0.113

### Validation of inflammation-based prognostic scores for CSS

In the multivariate analysis of the GPS model, pStage (HR, 6.624; 95% CI, 2.973–14.757; *p* < 0.001), CEA (HR, 2.162; 95% CI, 1.165–4.014; *p* = 0.015), GPS 1 (HR; 2.095, 95% CI; 1.025–4.283; *p* = 0.043), and GPS 2 (HR, 2.812; 95% CI, 1.111–7.116; *p* = 0.029) were identified as independent prognostic factors for CSS. In the mGPS model, pStage (HR, 6.941; 95% CI, 3.098–15.549; *p* < 0.001), and CEA (HR, 2.207; 95% CI, 1.186–4.107; *p* = 0.013) were identified as independent prognostic factors for CSS. Univariate analyses of the mGPS model revealed that an mGPS of 2 was a significant indicator of unfavorable CSS (*p* < 0.001); however, it was not confirmed to be an independent prognostic factor (*p* = 0.074) in multivariate analysis. Similarly, univariate analyses in the HS-mGPS model showed that pStage (HR, 7.063; 95% CI, 3.182–15.678; *p* < 0.001): and CEA (HR, 2.300; 95% CI, 1.236–4.279; *p* = 0.009) were identified as independent prognostic factors for CSS. An HS-mGPS of 2 was a significant indicator of unfavorable CSS (*p* < 0.001); however, it was not confirmed to be an independent prognostic factor (*p* = 0.080) in multivariate analysis (Table 2).

**Table 2:** Univariate and multivariate analyses of clinicopathological factors affecting CSS after laparoscopic gastrectomy for gastric cancer

	Variables	Univariate analysis			Multivariate analysis with GPS			Multivariate analysis with mGPS			Multivariate analysis with HS-mGPS		
		HR	95% CI	*p* value	HR	95% CI	*p* value	HR	95% CI	*p* value	HR	95% CI	*p* value
Age	< 70	1.000											
	≥ 70	1.072	0.592–1.940	0.820									
Gender	female	1.000											
	Male	1.573	0.777–3.185	0.208									
BMI	≥ 18.5	1.000											
	< 18.5	1.636	0.691–3.871	0.263									
Tumor size	< 5	1.000											
	≥ 5	3.848	2.013–7.356	< 0.001	1.074	0.484–2.385	0.860	1.142	0.511–2.553	0.746	1.105	0.491–2.485	0.809
Diff.	well & mod	1.000											
	Poor	1.899	1.041–3.465	0.037	1.519	0.783–2.948	0.216	1.565	0.809–3.027	0.184	1.521	1.521–2.923	0.209
pSstage	1 & 2	1.000											
	3	9.778	5.002–19.113	< 0.001	6.624	2.973–14.757	<0.001	6.941	3.098–15.549	< 0.001	7.063	3.182–15.678	< 0.001
CEA	< 5.0	1.000											
	≥ 5.0	2.745	1.511–4.987	< 0.001	2.162	1.165–4.014	0.015	2.207	1.186–4.107	0.013	2.300	1.236–4.279	0.009
GPS	0	1.000											
	1	2.308	1.188–4.485	0.014	2.095	1.025–4.283	0.043						
	2	6.442	3.087–13.441	< 0.001	2.812	1.111–7.116	0.029						
mGPS	0	1.000						1.000					
	1	2.258	0.698–7.302	0.174									
	2	6.442	3.087–13.441	< 0.001				2.241	0.926–5.426	0.074			
HS-mGPS	0	1.000									1.000		
	1	1.253	0.530–2.965	0.607									
	2	5.120	2.585–10.142	< 0.001							2.040	0.917–4.535	0.080
Complication	Absent	1.000											
	Present	1.802	0.981–3.310	0.058									

### OS analysis stratified by inflammation-based prognostic scores

**Figure 2:**
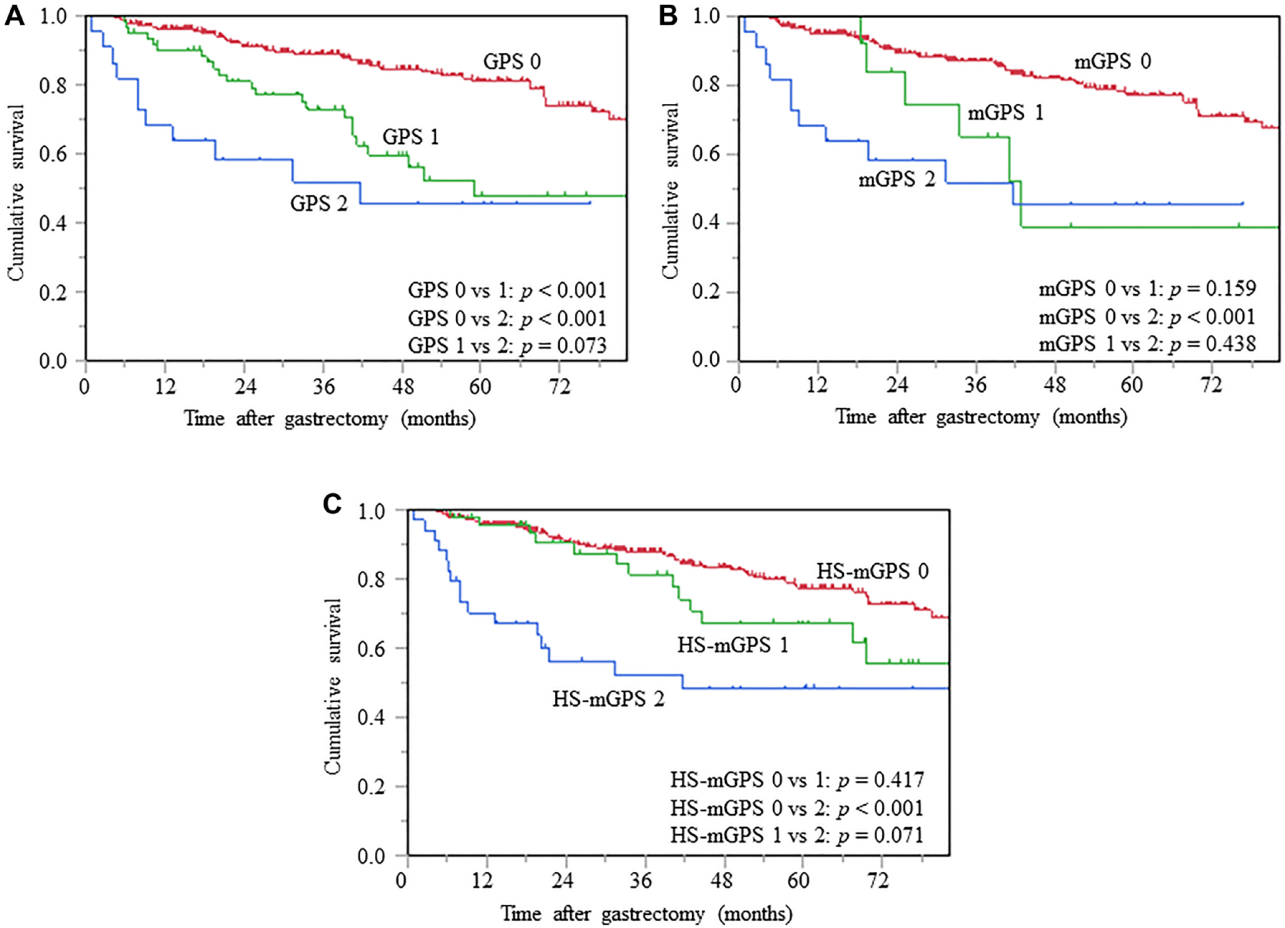
Kaplan-Meier curves of OS based on GPS (**A**), mGPS (**B**), and HS-mGPS (**C**), OS, overall survival; GPS, Glasgow prognostic score; mGPS, modified GPS; HS-mGPS, high-sensitivity mGPS.

The Kaplan-Meier survival analysis was conducted to evaluate the differences in prognostic impact of the GPS, mGPS, and HS-mGPS on OS, and the log-rank tests demonstrated significant differences in OS among the GPS 0 vs. 1 *(p* < 0.001) and 0 vs. 2 (*p* < 0.001) (Figure 2A). Patients with an mGPS of 2 showed a significantly unfavorable OS compared to those with an mGPS of 0 (*p* < 0.001) (Figure 2B). Patients with HS-mGPS 2 had a significantly worse OS than those with HS-mGPS 0 (*p* < 0.001) (Figure 2C).

### CSS analysis stratified by inflammation-based prognostic scores

**Figure 3:**
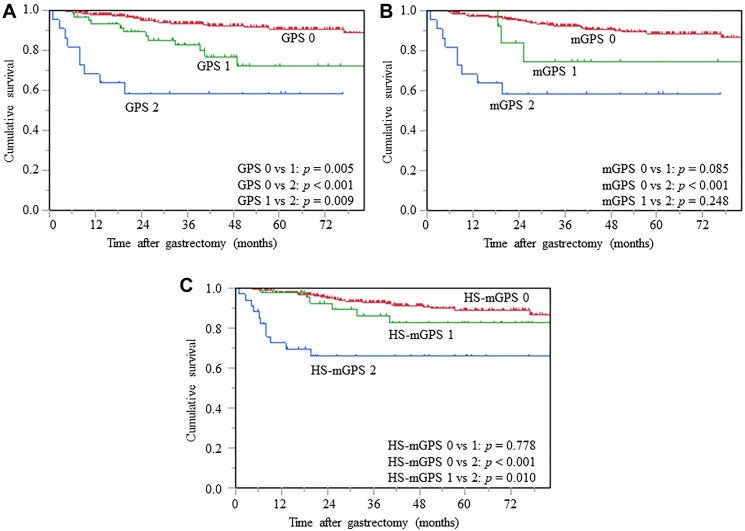
Kaplan-Meier curves of CSS based on GPS (**A**), mGPS (**B**), and HS-mGPS (**C**), CSS, cancer-specific survival; GPS, Glasgow prognostic score; mGPS, modified GPS; HS-mGPS, high-sensitivity mGPS.

The log-rank tests revealed significant differences in CSS among each of the 3 GPS (0 vs. 1; *p* = 0.005, 0 vs. 2; *p* < 0.001, 1 vs. 2; *p* = 0.009) (Figure 3A). Patients with mGPS 2 demonstrated significantly unfavorable CSS, compared to those with mGPS 0 (*p* < 0.001); however, there were no significant differences in mGPS 0 vs. 1 (*p* = 0.085) and GPS 1 vs. 2 (*p* = 0.248) (Figure 3B). Patients with HS-mGPS 2 had significantly worse CSS than those with HS-mGPS 0 (*p* < 0.001) and 1 (*p* = 0.010) (Figure 3C).

## DISCUSSION

Cancer-related inflammation leads to alteration of the tumor microenvironment and contributes to the promotion of cancer cell proliferation, invasion, and metastatic spread, and the inhibition of apoptosis and immunosuppression [16, 17]. Moreover, increasing evidence suggests that inflammation plays a role in cancer development and may also be accelerated by the cancer itself due to increased catabolism and malnutrition [18, 19]. CRP, a reliable marker of systemic inflammation, reflects cell-mediated immunity associated with poor outcomes in several cancers [3, 4]. Additionally, serum albumin levels are a leading indicator of nutritional status and serum albumin levels likely decrease secondarily to a systemic inflammatory response [1, 2]. Therefore, nutritional status and inflammatory response should be considered in conjunction when assessing the prognoses of cancer patients. Many studies seeking effective prognostic factors that could facilitate risk-stratified patient management and improvement of therapeutic outcomes are ongoing.

Forrest et al. [5] initially proposed the GPS, a composite of serum CRP and albumin levels, as a prognostic indicator for cancer patients, based on the concept that elevated CRP levels (cut-off, 1.0 mg/dl) or decreased albumin levels (cut-off, 3.5 mg/dl) may indicate an aggressive cancer progression. Subsequently, McMillan et al. [11] revised the GPS to the mGPS, a modified cumulative prognostic score, wherein patients with normal CRP levels are assigned a score of 0, irrespective of serum albumin levels, with the aim to predict the prognoses of various cancers more accurately. However, the ability of mGPS to predict poor prognoses is restricted because only a few patients show abnormal mGPS, when a cut-off value of 1.0 mg/dl for CRP is used [20–22]. Subsequent studies further refined the mGPS to the HS-mGPS using a further lower threshold for CRP (cut-off value: 0.3 mg/dl), in order to enhance the predictive ability of inflammation-based prognostic systems in cancer patients [15]. In this study, 83 (19.1%) and 36 (8.3%) patients with gastric cancer had abnormal scores of 1 or 2, respectively, according to the GPS and mGPS scoring systems. However, when a lower cut-off value for CRP in the HS-mGPS scoring system was used, 82 (18.9%) patients had abnormal scores, which was a more sensitive result than that of mGPS and equal to that of GPS. The impact of HS-mGPS on the long-term prognosis of gastric cancer patients is not well understood, and this is one of the first reports to study this aspect, but it did not show a superiority of HS-mGPS.

In this retrospective cohort study, we analyzed three inflammation-based prognostic scoring systems, namely GPS, mGPS, and HS-mGPS, to evaluate which of these is the strongest predictor of long-term survival of patients with gastric cancer. Resultantly, the original GPS scoring system showed the largest AUC, compared to the AUCs of the mGPS and HS-mGPS, suggesting that the GPS has the best ability to predict OS and CSS in patients with gastric cancer. The log-rank tests for the GPS revealed significant differences in OS, in the comparison of scores 0 vs. 1, and scores 0 vs. 2; however, in the mGPS and HS-mGPS systems, the corresponding difference was observed only in the comparison of score 0 vs. 2. Moreover, each GPS was independently associated with CSS. In addition, among the three inflammation-based prognostic scoring systems, GPS alone was found to be an independent prognostic predictor of CSS in multivariate analysis. Thus, compared to its derivatives, the original GPS appeared to be a more useful predictor of long-term survival in patients with gastric cancer. This may be because the GPS is determined using both CRP and albumin values, whereas scores of 0 in the mGPS and HS-mGPS systems are determined by CRP alone, regardless of albumin levels [5, 11]. That is, although hypoalbuminemia is more likely to occur secondary to elevated CRP levels, a crucial difference between the GPS and mGPS is the inclusion of patients with hypoalbuminemia in the absence of elevated CRP levels. This means that both the inflammatory response and nutritional status must be taken into account, in order to predict the prognoses of cancer patients more accurately.

The GPS was first presented as a scoring system based on a combination of CRP and albumin in patients with inoperable non-small cell lung cancer [5]. In gastric cancer, Crumley et al. reported that the GPS is a CSS predictor in patients with inoperable gastroesophageal cancer [23]. The GPS was usually considered to correlate with postoperative survival only in very advanced cancers. However, Kubota et al. analyzed gastric cancer patients undergoing gastrectomy to evaluate the prognostic ability of the mGPS in very advanced, as well as relatively early gastric cancer [24]. In this study, a significant survival difference was observed even in patients with relatively early stage I gastric cancer, which suggests that the mGPS is not only a prognostic tool for very advanced gastric cancer, but also for relatively early-stage gastric cancer. This suggests that the mGPS may be a prognostic factor for survival in patients with relatively early-stage gastric cancer. On the other hand, in patients undergoing surgery for colorectal cancer, preoperative mGPS has been independently reported to be associated with an increased risk of developing postoperative infectious complications, while others reported that it is not associated with gastric cancer [25, 26]. Thus, GPS and its derivatives may have different clinical significances in different cancers.

This study has several limitations. First, selection bias may be present because this was a single-institution, retrospective study. Second, we focused on the preoperative assessment of the GPS and its derivatives, without evaluating their postoperative changes. Third, the other parameters correlating systemic inflammation and nutrition, such as neutrophil-to-lymphocyte ratio, systemic inflammatory index, and prognostic nutritional index, were not evaluated. It remains debatable whether the prognoses of cancer patients can be improved by nutritional intervention. Further studies are warranted to address the above-mentioned limitations.

In conclusion, our results indicated that GPS is the most reliable assessment tool among the three assessed inflammation-based prognostic scoring systems for predicting long-term survival of patients with gastric cancer who underwent curative laparoscopic gastrectomy. Further studies on gastric cancer and other malignancies, and prospective multi-institutional studies are needed to clarify the efficacy of GPS and its derivatives, which will further enable beneficial clinical applications.

## MATERIALS AND METHODS

### Patients

We retrospectively reviewed the medical records of 434 consecutive patients with histologically verified gastric adenocarcinoma who underwent curative laparoscopic gastrectomy at our institution between January 2010 and December 2017.

The exclusion criteria included patients with an inflammatory, bone marrow, hematological, or autoimmune disease; patients who underwent neoadjuvant chemotherapy; patients with an active infection within one month before surgery; and patients with histories of other malignancies within the preceding 5 years.

The extent of gastrectomy and lymph node dissection were determined in accordance with the Japanese Gastric Cancer Treatment Guidelines (version 4) [27]. The clinicopathological classification of gastric cancer was assessed per the International Union Against Cancer Tumor, Node, Metastasis (TNM) classification (seventh edition) [28]. Postoperative complications were classified according to the Clavien-Dindo (CD) grading system, and those with CD grade II or higher complications were assessed [29].

The retrospective protocol of this study was approved by the Ethical Review Board of Shimane University, Faculty of Medicine (Shimane, Japan), and the study is registered with the University Hospital Medical Information Network Clinical Trials Registry (UMIN000030472). The requirement for informed consent was waived because of the retrospective nature of this cohort study.

### Criteria of systemic inflammation-based prognostic scores

Preoperative laboratory data and physical measurements were obtained from each patient within 7 days before surgery. The GPS was scored by allocating one point each for elevated CRP (> 1.0 mg/dl) and hypoalbuminemia (< 3.5 mg/dl), and the patients with both, either, or none of these laboratory parameters were assigned scores of 2, 1, or 0, respectively [5]. For the mGPS, patients with an elevated CRP (> 1.0 mg/dl) and hypoalbuminemia (< 3.5 mg/dl) were assigned a score of 2; those with an elevated CRP alone, a score of 1; and those with a normal CRP regardless of the albumin levels, a score of 0 [11]. The HS-mGPS was calculated based on the cut-off values of 0.3 mg/dl for CRP and 3.5 g/dl for albumin levels [15]. Patients with an elevated CRP (> 0.3 mg/dl) and hypoalbuminemia (< 3.5 mg/dl) were assigned a score of 2, those with an elevated CRP alone were assigned a score of 1, and those with hypoalbuminemia alone or without any abnormal values were assigned a score of 0 (Table 3).

**Table 3:** Criteria for systemic inflammation-based prognostic score

Prognostic marker	Criteria	Score
GPS	CRP ≤ 1.0 mg/dl and Alb ≥ 3.5 mg/dl	0
	CRP > 1.0 mg/dl or Alb < 3.5 mg/dl	1
	CRP > 1.0 mg/dl and Alb < 3.5 mg/dl	2
mGPS	CRP ≤ 1.0 mg/dl	0
	CRP > 1.0 mg/dl and Alb ≥ 3.5 mg/dl	1
	CRP > 1.0 mg/dl and Alb < 3.5 mg/dl	2
HS-mGPS	CRP ≤ 0.3 mg/dl	0
	CRP > 0.3 mg/dl and Alb ≥ 3.5 mg/dl	1
	CRP > 0.3 mg/dl and Alb < 3.5 mg/dl	2

### Postoperative follow-up programs

After undergoing surgery, patients were carefully followed up by physical examination and blood tests, every 3 months, for 5 years. Computed tomography scans were performed every 6 months for 3 years, and then every year from year 3 to year 5. Adjuvant chemotherapy using 5-fluorouracil-based adjuvant chemotherapy regimens (cisplatin plus tegafur/gimeracil/oteracil or capecitabine) was administered to the majority of patients with advanced gastric cancer. Furthermore, post recurrence chemotherapy was administered according to the guidelines edited by the Japan Gastric Cancer Association [27]. OS was calculated from the date of gastrectomy to the date of either death or last follow-up. CSS was defined as the interval from the date of surgery and to the date of cancer-specific death.

### Statistical analysis

The Chi-squared or Fisher’s exact tests were used to evaluate the differences between the categorical variables. The OS and CSS were assessed using the Kaplan-Meier method and compared using the log-rank test. Cox proportional hazards regression models were used to estimate the HR with 95% CI. All statistical analyses were carried out using the JMP software (version 15 for Windows; SAS Institute). A *p*-value of < 0.05 was considered statistically significant. The time-dependent ROC curves were generated and the AUCs were calculated to evaluate the discriminatory ability of each scoring system.

## SUPPLEMENTARY MATERIALS





## Abbreviations

AUC: area under the curve
CEA: carcinoembryonic antigen
CI: confidence interval
CD: Clavien-Dindo
CRP: C-reactive protein
CSS: cancer-specific survival
GPS: Glasgow prognostic score
HR: hazard ratio
HS-mGPS: high-sensitivity mGPS
MBI: body mass index
mGPS: modified GPS
OS: overall survival
pStage: pathological stage
ROC: Receiver operating characteristic
TNM: Tumor, Node, Metastasis
