# Supplementary material for: Glasgow prognostic score is a better predictor of the long-term survival in patients with gastric cancer, compared to the modified Glasgow prognostic score or high-sensitivity modified Glasgow prognostic score
Source: Oncotarget. 2020 Nov 10;11(45):4169–77. doi: 10.18632/oncotarget.27796 (PMC7665228; doi:10.18632/oncotarget.27796)
Supplement: Supplementary file 1 [file oncotarget-11-4169-s001.pdf]

Glasgow prognostic score is a better predictor of the long-term survival in patients with gastric cancer, compared to the modified Glasgow prognostic score or high-sensitivity modified Glasgow prognostic score

SUPPLEMENTARY MATERIALS

Supplementary Table 1: Associations of clinical characteristics with GPS, mGPS, and HS-mGPS.
See Supplementary Table 1
