# Supplementary material for: Glasgow prognostic score is a better predictor of the long-term survival in patients with gastric cancer, compared to the modified Glasgow prognostic score or high-sensitivity modified Glasgow prognostic score
Source: Oncotarget. 2020 Nov 10;11(45):4169–77. doi: 10.18632/oncotarget.27796 (PMC7665228; doi:10.18632/oncotarget.27796)
Supplement: Supplementary file 2 [file oncotarget-11-4169-s002.docx]

**Supplementary Table 1: Associations of clinical characteristics with GPS, mGPS, and HS-mGPS**

|  | **Characteristics** | | **Number of patients** | **GPS** | | | |  | **mGPS** | | | |  | **HS-mGPS** | | | |
| --- | --- | --- | --- | --- | --- | --- | --- | --- | --- | --- | --- | --- | --- | --- | --- | --- | --- |
|  |  |  |  | **0** | **1** | **2** |  |  | **0** | **1** | **2** |  |  | **1** | **2** | **3** |  |
|  |  |  |  | **(*n* = 351)** | **( *n* = 61)** | **(*n* = 22)** | ***p* value** |  | **(*n* = 398)** | **(*n* = 14)** | **(*n* = 22)** | ***p* value** |  | **(*n* = 352)** | **(*n* = 48)** | **(*n* = 34)** | ***p* value** |
|  | Age (years old) | |  |  |  |  | 0.005 |  |  |  |  | 0.021 |  |  |  |  | 0.022 |
|  |  | < 70 | 201 | 175 | 21 | 5 |  |  | 192 | 4 | 5 |  |  | 173 | 19 | 9 |  |
|  |  | ≥ 70 | 233 | 176 | 40 | 17 |  |  | 206 | 10 | 17 |  |  | 179 | 29 | 25 |  |
|  | Sex | |  |  |  |  | 0.156 |  |  |  |  | 0.257 |  |  |  |  | 0.176 |
|  |  | Male | 303 | 238 | 48 | 17 |  |  | 274 | 12 | 17 |  |  | 239 | 37 | 27 |  |
|  |  | Female | 131 | 113 | 13 | 5 |  |  | 124 | 2 | 5 |  |  | 113 | 11 | 7 |  |
|  | BMI | |  |  |  |  | 0.010 |  |  |  |  | 0.745 |  |  |  |  | 0.214 |
|  |  | ≥ 18.5 | 39 | 24 | 12 | 3 |  |  | 35 | 1 | 3 |  |  | 30 | 3 | 6 |  |
|  |  | < 18.5 | 395 | 327 | 49 | 19 |  |  | 363 | 13 | 19 |  |  | 322 | 45 | 28 |  |
|  | Albumin (g/dl) | |  |  |  |  | < 0.001 |  |  |  |  | < 0.001 |  |  |  |  | < 0.001 |
|  |  | < 3.5 | 366 | 351 | 15 | 0 |  |  | 352 | 14 | 0 |  |  | 318 | 48 | 0 |  |
|  |  | ≥ 3.5 | 68 | 0 | 46 | 22 |  |  | 46 | 0 | 22 |  |  | 34 | 0 | 34 |  |
|  | CRP (mg/l) | |  |  |  |  | < 0.001 |  |  |  |  | < 0.001 |  |  |  |  | < 0.001 |
|  |  | ≤0.3 | 352 | 317 | 35 | 0 |  |  | 352 | 0 | 0 |  |  | 352 | 0 | 0 |  |
|  |  | 0.3< & ≤1.0 | 46 | 34 | 12 | 0 |  |  | 46 | 0 | 0 |  |  | 0 | 34 | 12 |  |
|  |  | >1.0 | 36 | 0 | 14 | 22 |  |  | 0 | 14 | 22 |  |  | 0 | 14 | 22 |  |
|  | Location of tumor | |  |  |  |  | 0.131 |  |  |  |  | 0.190 |  |  |  |  | 0.250 |
|  |  | EGJ | 11 | 10 | 0 | 1 |  |  | 10 | 0 | 1 |  |  | 10 | 0 | 1 |  |
|  |  | U | 85 | 63 | 13 | 9 |  |  | 73 | 3 | 9 |  |  | 64 | 10 | 11 |  |
|  |  | M | 184 | 151 | 26 | 7 |  |  | 169 | 8 | 7 |  |  | 155 | 17 | 12 |  |
|  |  | L | 154 | 127 | 22 | 5 |  |  | 146 | 3 | 5 |  |  | 123 | 21 | 10 |  |
|  | Procedure | |  |  |  |  | 0.013 |  |  |  |  | 0.021 |  |  |  |  | 0.007 |
|  |  | LTG | 91 | 65 | 15 | 11 |  |  | 77 | 3 | 11 |  |  | 65 | 11 | 15 |  |
|  |  | LPG | 44 | 36 | 5 | 3 |  |  | 40 | 1 | 3 |  |  | 36 | 3 | 5 |  |
|  |  | L(A)DG | 299 | 250 | 41 | 8 |  |  | 281 | 10 | 8 |  |  | 251 | 34 | 14 |  |
|  | Tumor size (mm) | |  |  |  |  | < 0.001 |  |  |  |  | < 0.001 |  |  |  |  | < 0.001 |
|  |  | < 5 | 259 | 229 | 26 | 4 |  |  | 245 | 10 | 4 |  |  | 226 | 28 | 5 |  |
|  |  | ≥ 5 | 175 | 122 | 35 | 18 |  |  | 153 | 4 | 18 |  |  | 126 | 20 | 29 |  |
|  | Tumor differentiation | | |  |  |  | 0.506 |  |  |  |  | 0.625 |  |  |  |  | 0.471 |
|  |  | Well | 81 | 70 | 9 | 2 |  |  | 77 | 2 | 2 |  |  | 68 | 9 | 4 |  |
|  |  | Moderate | 162 | 127 | 24 | 11 |  |  | 145 | 6 | 11 |  |  | 125 | 20 | 17 |  |
|  |  | Poor | 191 | 154 | 28 | 9 |  |  | 176 | 6 | 9 |  |  | 159 | 19 | 13 |  |
|  | Depth of tumor | |  |  |  |  | < 0.001 |  |  |  |  | < 0.001 |  |  |  |  | < 0.001 |
|  |  | T1a-1b | 232 | 207 | 21 | 4 |  |  | 223 | 5 | 4 |  |  | 204 | 22 | 6 |  |
|  |  | 2 | 56 | 49 | 6 | 1 |  |  | 55 | 0 | 1 |  |  | 45 | 7 | 4 |  |
|  |  | 3 | 58 | 42 | 13 | 3 |  |  | 53 | 2 | 3 |  |  | 47 | 6 | 5 |  |
|  |  | 4a-4b | 88 | 53 | 21 | 14 |  |  | 67 | 7 | 14 |  |  | 56 | 13 | 19 |  |
|  | Lymph node metastasis | | |  |  |  | 0.023 |  |  |  |  | 0.015 |  |  |  |  | 0.017 |
|  |  | N0 | 284 | 242 | 33 | 9 |  |  | 269 | 6 | 9 |  |  | 245 | 27 | 14 |  |
|  |  | N1 | 48 | 39 | 5 | 4 |  |  | 44 | 0 | 4 |  |  | 37 | 6 | 6 |  |
|  |  | N2 | 53 | 35 | 13 | 5 |  |  | 44 | 4 | 5 |  |  | 37 | 6 | 10 |  |
|  |  | N3 | 49 | 35 | 10 | 4 |  |  | 41 | 4 | 4 |  |  | 36 | 9 | 4 |  |
|  | Pathological TNM stage | | |  |  |  | < 0.001 |  |  |  |  | < 0.001 |  |  |  |  | < 0.001 |
|  |  | 1a-1b | 260 | 230 | 26 | 4 |  |  | 251 | 5 | 4 |  |  | 226 | 26 | 8 |  |
|  |  | 2a-2b | 69 | 54 | 11 | 4 |  |  | 64 | 1 | 4 |  |  | 55 | 8 | 6 |  |
|  |  | 3a-3c | 105 | 67 | 24 | 14 |  |  | 83 | 8 | 14 |  |  | 71 | 14 | 20 |  |
|  | CEA antigen (ng/ml) | | |  |  |  | 0.062 |  |  |  |  | 0.048 |  |  |  |  | 0.122 |
|  |  | < 5.0 | 333 | 274 | 47 | 12 |  |  | 309 | 12 | 12 |  |  | 274 | 38 | 21 |  |
|  |  | ≥ 5.0 | 101 | 77 | 14 | 10 |  |  | 89 | 2 | 10 |  |  | 78 | 10 | 13 |  |
|  | Postoperative complications | | |  |  |  | < 0.001 |  |  |  |  | < 0.001 |  |  |  |  | <0.001 |
|  |  | Absent | 306 | 262 | 38 | 6 |  |  | 293 | 7 | 6 |  |  | 258 | 36 | 12 |  |
|  |  | Present | 128 | 89 | 23 | 16 |  |  | 105 | 7 | 16 |  |  | 94 | 12 | 22 |  |
